# Religious Newspapers and Quackery

**Published:** 1888-07

**Authors:** L. P. Gibson

**Affiliations:** Sec’y.; Little Rock, Ark.


### RELIGIOUS NEWSPAPERS AND QUACKERY.

---

*Editors Atlanta Medical and Surgical Journal:*

In compliance with instructions I transmit herewith the following Resolutions adopted at the Thirteenth Annual Session of the State Medical Society of Arkansas, held at Fort Smith, April 25, 26 and 27, 1888, and ordered to be furnished to the American Medical Association, the medical and religious press, and to the State Medical Societies, soliciting their co-operation in bringing about a correction of these grievous and palpable errors:

*Resolved*, That the members of the State Medical Society of Arkansas have for years observed with pain and mortification the patronage given to charlatanism in all its multifarious aspects by the religious press of our country.

*Resolved further and most specifically*, That the appearance in religious papers, ostensibly published for the inculcation of truth and morality, of serious homilies on prayer and praise side by side with cures for consumption, cancer, Bright's disease and other incurable ailments, to which an editorial endorsement is often given, as well as secret preparations under the cloak of remedies for disease, but really intended for purposes of fœticide and other immoral uses, largely tends to shake the confidence of the profession of medicine in the integrity and purpose of the managers and editors of such journals.

*Resolved further*, That it has been the well-known custom of the profession to render services gratuitously to clergymen, which we do not regret, nor do we propose to recall, yet we must assert that the frequent occurrence of endorsements and recommendations of the clergy of pêripatetic doctors and advertising charlatans has in many instances been the only reward of our gratuitous services.

*Resolved further*, That we are aware that the editors of religious newspapers admit the painful situation in which these advertisements place them, and attempt to excuse themselves by saying that it is necessary to take these advertisements in order to obtain means to conduct their papêrs; but, in the language of orthodox theology, we would say: "Put behind you that damnable doctrine that we must do evil that good may come."

*Resolved further*, That, as a Society, we declare that the continued perpetration of the above offenses by some of the clergy and religious press brings harm to the bodies of their constituency, and damages materially their influence upon the thinking class of the medical profession.

*Resolved*, That the Secretary be instructed to furnish copies of these resolutions to the religious and medical press of the United States, to the American Medical Association and to the State Medical Societies, soliciting their co-operation in bringing about a correction of these grievous and palpable errors.

Very respectfully,

L. P. GIBSON, M. D., Sec'y.

*Little Rock, Ark., May 15, 1888.*

